# Reducing the risk of age-related macular degeneration progression - five-year follow-up study in Ukraine and Moldova

**DOI:** 10.22336/rjo.2025.55

**Published:** 2025

**Authors:** Olga Vladimirovna Guzun, Oleg Serhiyovich Zadorozhnyy, Nataliya Valerievna Konovalova, Pavel Andriyovych Bezdetko, Andrii Rostyslavovich Korol, Lilia Gheorghe Dumbrăveanu, Valeriu Nicon Cușnir, Vitalie Valeriu Cușnir

**Affiliations:** 1 SI “The Filatov Institute of Eye Diseases and Tissue Therapy of the National Academy of Medical Sciences of Ukraine”, Odesa, Ukraine; 2 Odesa National Medical University, Odesa, Ukraine; 3 Kharkiv National Medical University, Kharkiv, Ukraine; 4 “Nicolae Testemiţanu” State University of Medicine and Pharmacy of the Republic of Moldova, Chişinău, Moldova

**Keywords:** age-related macular degeneration, Nutrof®Forte, photobiomodulation, drusen, best-corrected visual acuity, AMD = age-related macular degeneration, RPE = retinal pigment epithelium, PBM = photobiomodulation, PUFAs = omega-3 polyunsaturated fatty acids, SER = spherical equivalent refraction, IOP = intraocular pressure, BCVA = best-corrected visual acuity, HR = hazard ratio

## Abstract

This open prospective study aimed to evaluate the dynamics of progression of early and intermediate age-related macular degeneration (AMD) against the background of continuous use of the nutraceutical formula AREDS2, which includes omega-3 polyunsaturated fatty acids (PUFAs), vitamin D, resveratrol, and photobiomodulation (PBM), over a 5-year follow-up in patients from Ukraine and Moldova. Examining 126,400 patients, 163 patients (304 eyes) with early and intermediate stages of AMD were treated (5-year follow-up).

Patients were divided into two groups. Patients in the 1st group (149 eyes) were prescribed a nutraceutical formula based on AREDS2 with omega-3 PUFAs, vitamin D, and resveratrol (Nutrof®Forte 1 capsule once a day continuously). The second group (155 eyes) included patients who irregularly took various vitamin-antioxidant complexes. All patients underwent PBM every 6 months.

The five-year prevalence of early and intermediate AMD was estimated using data from leading ophthalmological centers in Ukraine (Odesa - 7.1%, Kharkiv - 6.6%) and Moldova (6.3%).

AMD progression in the multivariate Cox regression model over five years showed a 3.24-fold reduction in relative risk (95% CI: 2.15-4.79, p=0.000) for patients with early and intermediate AMD who regularly took the recommended nutraceutical (compared to those who irregularly took various vitamin-antioxidant complexes).

Patients with early and intermediate AMD are recommended to undergo courses of PBM every six months. Additionally, it is crucial to address cardiovascular issues and consistently use the AREDS2 nutraceutical formula. Adherence to these recommendations can reduce the likelihood of disease progression by at least 3.24 times over the next 5 years.

## Introduction

Age-related macular degeneration (AMD) is a progressive neurodegeneration of photoreceptors, retinal pigment epithelium (RPE), and Bruch’s membrane, resulting in loss of central vision with a projected global prevalence of 288 million by 2040 [1]. In European countries, the prevalence ranges from 12.3% to 18.3% [2]. In Asia, the prevalence is 7.38%, in Africa, 7.53%, and in China, 4.9% [3]. This increase in prevalence raises the need for effective treatment of AMD, which, given the incurable nature of the disease, should be permanent.

AMD is a multifactorial disease, with the main risk factors being age and heredity (genetic polymorphisms in AMD account for 46-71%) [4]. Cardiovascular disease and hyperlipidemia increase the risk for the development and progression of AMD [5]. Cellular ageing with age leads to changes in Bruch’s membrane permeability, accumulating in the metabolic products in the retina. Apoptosis, pyroptosis, and necroptosis are involved in the death of RPE cells in AMD [6]. Even short-term effects of photooxidative damage include immune cell activation and photoreceptor cell death in the retina and contribute to the progression of AMD [7]. Many authors use photobiomodulation (PBM) [6,8**-**10] for effective cellular defense against oxidative stress and prevention of disease progression, as well as antioxidants [11]. Consequently, the combined use of PBM courses and continuous use of the nutraceutical formula AREDS2 with omega-3 polyunsaturated fatty acids (PUFAs), vitamin D, and resveratrol with high antioxidant activity may be a promising therapeutic strategy for ocular diseases associated with aging. Consequently, we hypothesized that comprehensive and regular treatment of patients with early/intermediate-stage AMD may slow the progression of the disease to later stages.

The study aimed to evaluate the dynamics of progression of early and intermediate age-related macular degeneration against the background of continuous use of the nutraceutical formula AREDS2, which includes omega-3 PUFAs, vitamin D, resveratrol, and photobiomodulation, over a 5-year follow-up in patients from Ukraine and Moldova.

## Material and methods

### Study design and participants

The epidemiological data of all patients (n = 126,400) who sought ophthalmological care between 2019 and 2023 in highly specialized centers of large cities in Ukraine (Kharkiv, Odesa) and Moldova were analyzed. Furthermore, an open prospective study was conducted based on the SI “The Filatov Institute of Eye Diseases and Tissue Therapy of the National Academy of Medical Sciences of Ukraine”, involving 163 patients (304 eyes) with early and intermediate forms of AMD. Data were collected over five years during the study (T_0_, T_1_, T_2_, T_3_, T_4_, T_5_).

The Bioethics Committee of the Institute accepted this study conducted in accordance with the Declaration of Helsinki (protocol No. 23 of 01.05.2024). Written informed consent was obtained from all patients.

All patients underwent standard ophthalmological examination, including refractometry, tonometry, determination of maximum corrected visual acuity (BCVA), biomicroscopy, ophthalmoscopy of the ocular fundus, and optical coherence tomography (OCT).

*Inclusion criteria* involved patients over 50 years of age with eyes diagnosed with early or intermediate AMD according to the classification [12]. Early AMD included medium-sized drusen measuring >63 μm and ≤125 μm, with no RPE abnormalities. Intermediate AMD involved large drusen >125 μm and/or any RPE abnormalities associated with drusen (AREDS3). Participants with a minimum follow-up period of 3 years were included.

*Exclusion criteria* were represented by patients with late AMD or the presence of any other pathology on baseline or follow-up OCT, including macular disorders other than AMD (e.g., diabetic retinopathy, central serous chorioretinopathy, epiretinal membrane) and concomitant administration of different medications. Additional exclusion criteria were prior intraocular treatment (e.g., intravitreal injections, laser photocoagulation, micropulse laser), prior intraocular surgery (excluding uncomplicated cataract surgery). If both eyes of a patient met the study criteria, they were both included in the study.

*AMD progression* was defined as a change in disease stage from early to intermediate stage AMD, and the presence of signs of late-stage disease in eyes previously classified as early or intermediate stage. AMD progression was not determined when none of the signs of later-stage disease were observed.

*Statistical analysis* of data was performed using Statistical software (version 10.0, StatSoft Inc., USA). Qualitative data were described with numbers and percentages using frequency tables. Quantitative variables were tested for normal distribution using the Shapiro-Wilk test. Parametric data were presented as mean ± standard deviation and 95% CI (M±SD [95% CI]), and nonparametric data were presented as median (Me) and 25% and 75% quartiles (Q 25%-75%). An even Student’s t-test was used to compare the two parametric variables. Nonparametric data were compared between groups using the Mann-Whitney U-criterion, and these data were analyzed according to AMD progression using the Kruskal-Wallis rank criterion. The χ^2^ or Fisher’s exact test was used to analyze categorical data. Spearman correlation analysis (r_s_) was used. Cox proportional-hazards regression analysis was performed to identify a set of independent predictors of AMD progression, followed by inclusion of significant predictors in stepwise multivariate Cox regression. Significant signs from the single-factor regression results were included as covariates in the stepwise regression equation. A dependent sign was a progression of AMD given the treatment group. The relative risk and 95% confidence interval of endpoint development in the presence of the studied characteristic in different age categories were calculated. Values of p<0.05 were considered statistically significant.

### Clinical characteristics of patients with AMD

**Fig. 1:**
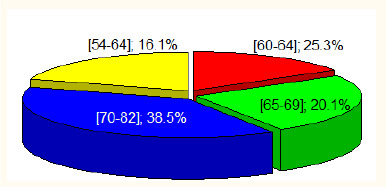
Distribution of patients with early and intermediate AMD according to their age

We examined and treated 163 patients (304 eyes) with early and intermediate AMD. In 22 patients (13%), only one eye with early AMD was included in the study; the other eye was diagnosed with age-related maculopathy (drusen < 63 μm). The mean age of the patients was 66.0 (60-70) years. There were 56 (34%) males in the study. In our study, patients with AMD were distributed by age as follows: in the age group from 54 to 59 years, 16.1%, 60 to 64 years, 25.3%, 65 to 69 years, 20.1%, 70 to 82 years, 38.5% (**Fig. 1**). One hundred five patients (64%) were of working age.

Two hundred twelve eyes (70%) were diagnosed with early-stage (AREDS1) and 92 eyes (30%) with intermediate-stage (AREDS2) AMD. Patients were divided into two groups: Group 1 - 79 patients (149 eyes) and Group 2 - 84 patients (155 eyes) (**Table 1**).

**Table 1:** Clinical characteristics of the study cohort (163 patients - 304 eyes)

Indicators before treatment	Group I, n = 149 eyes (79 patients)	II group, n = 155 eyes (84 patients)	p
Age (year)	61.0 (59-69)	70 (64-74)	p=0.01^a^
Men/women	27 (34%)/52 (66%)	29 (35%)/65 (56%)	p=0.74^c^
AMD stage: Early (AREDS1)/ Intermediate (AREDS2)	91 eyes (61.1%)/ 58 eyes (38.9%)	120 eyes (77.4%)/ 35 eyes (22.6%)	p=0.000^c^ p=0.02^c^
SER, D	0.32±0.88	0.46±0.96	p=0.2^c^
L, (mm)	23.3 (22.7-23.7)	23.2 (22.6-23.7)	p=0.96^a^
IOP (mm Hg)	15.0 (14.0-16.0)	15.0 (14.0-17.0)	p=0.56^a^
Cataract/ Artifakia	63 eyes (42.3%)/ 63 eyes (42.3%)	52 eyes (33.5%)/ 73 eyes (47.1%)	p=0.32^c^ p=0.61^c^
BCVA	0.5 (0.2-0.65)	0.6 (0.45-0.7)	p=0.003^a^
Central retinal thickness (μm)	231 (213-300)	209 (199-229)	p=0.000^a^
Central choroidal thickness (μm)	218 (212-256)	228 (209-244)	p=0.63^a^
Cardiovascular disease, yes.	37 patients (47%)	48 patients (57%)	p=0.39^c^
Smoking, yes	20 patients (25%)	19 patients (23%)	p=0.87^c^
Diabetes mellitus, type II	6 patients (7.5%)	5 patients (6%)	p=0.77^c^
Observation time, days	1430 (995; 2050)	995 (613; 1510)	p=0.000 ^a^

Note: p = significance level of the difference between the indicators: a = by Mann-Whitney test with Me (Q 25%-75%), c = by Fisher’s exact test with n (%), c = by Student’s t-criterion with M±SD (95% CI). SER = spherical equivalent refraction; IOP = intraocular pressure; BCVA = best-corrected visual acuity

A total of 136 eyes (44.7%) of all eyes examined were pseudophakic; primary age-related cataract was observed in 115 eyes (37.8%). The most commonly documented retinal changes detected on routine ophthalmologic examination were posterior hyaloid membrane detachment (25%), epiretinal membrane (17%), and glaucoma (14%). 11 patients (6.7%) had stabilized type II diabetes mellitus, which was detected at 3 and 4 years of follow-up. The minimum follow-up period was 3 years. In group 1, 50% of patients were observed for 4.3 years, and in group 2, for 3 years. There was a difference in the follow-up period between the groups. Due to the detected deterioration of the ocular fundus, these patients were prescribed other treatments. From the anamnesis, 39 patients (24%) were smokers. The frequency of systemic hypertension, cardiac arrhythmia, and smoking history did not differ between the two groups. A family physician examined all patients, and correction of concomitant cardiovascular pathology was prescribed. After examination by a family physician, 85 patients (52.1%) had cardiovascular pathology (64 patients (75.3%) had hypertension (HT) I-III degree, and 21 patients (24.7%) had chronic ischemic heart disease, arrhythmia (CHD) I-II degree).

### Treatment

All patients underwent photobiomodulation (PBM) of the retina every 6 months using diode laser SM-4.3 (λ=650 nm, W=0.4 mW/cm^2^, exposure 300 s, course 10 days). After the FBM course, no significant difference in the investigated indices between the groups was noted; therefore, the results of these data were not presented in the paper. Additionally, nearly all patients reported improvements in brightness, clarity of vision, and quality of life [8,9].

Group 1 patients were prescribed a nutraceutical formula AREDS2 with omega-3 PUFAs, vitamin D, and resveratrol (Nutrof®Forte 1 capsule once a day continuously) [13].

The second group included patients who were prescribed the same formula but took various vitamin-antioxidant complexes irregularly instead.

No side effects were noted in any patient during the entire follow-up period.

**Fig. 2:**
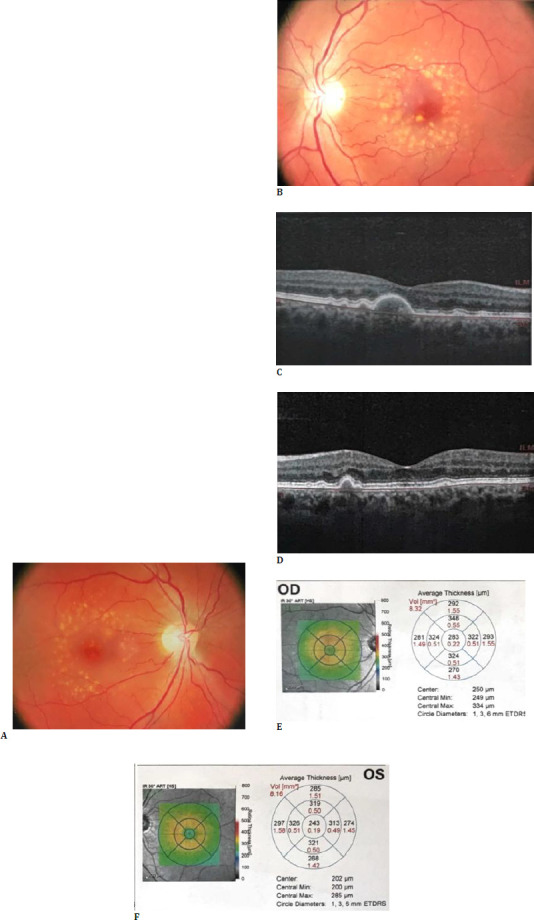
Patient K. 61 years old. **A, B**. Fundus image of both eyes. In the right eye, there are medium and large drusen, while in the left eye, medium-sized drusen are combined with RPE abnormalities. **C, D**. OCT of the macular area. In the macula of the right eye, the largest drusen exceeds 125 µm, while in the left eye, the drusen size is 125 µm or less. **E, F**. Central retinal thickness map of both eyes

Fundus imaging remains the most accurate method of AMD diagnosis and the characteristics of its stage and development. Control of morphological changes in the retina, RPE, and blood vessels using ophthalmoscopy, colour fundus photography, OCT, OCT-A, FAG are presented in **Fig. 2A-F**.

## Results

We analyzed the prevalence of AMD for five years (2019-2023) in Ukraine and Moldova. According to data from various ophthalmologic centers in Ukraine, 74,200 patients in Odesa and 52,200 patients in Kharkiv were examined. The prevalence of dry AMD over five years was in Odesa - 7.1% (5260/74200), in Kharkiv - 6.6% (3450/52200), and in Moldova - 6.3% (3240/51300) of patients.

The rate of change in BCVA (T_5_-T_0_) over five years of follow-up in both groups is presented in **Table 2**.

**Table 2:** Change rate of BCVA (T_5_-T_0)_ over five years of follow-up in both groups

	BCVA T_0_		BCVA T_1_		BCVA T_2_		BCVA T_3_		BCVA T_4_		BCVA T_5_	
	1	2	1	2	1	2	1	2	1	2	1	2
Valid	149	155	149	155	132	147	129	120	136	110	129	108
Mean	0.47	0.56	0.59	0.66	0.58	0.55	0.66	0.54	0.59	0.49	0.59	0.49
Minimum	0.10	0.10	0.17	0.10	0.08	0.10	0.10	0.08	0.10	0.08	0.10	0.08
Maximum	0.85	0.85	1.00	1.00	1.00	0.88	1.10	1.00	1.10	0.95	1.10	0.95
25th percentile	0.20	0.45	0.30	0.50	0.29	0.50	0.35	0.44	0.30	0.40	0.30	0.39
75th percentile	0.65	0.70	0.85	0.85	0.85	0.70	0.95	0.70	0.90	0.70	0.90	0.70

Mann-Whitney U test p=0.003 p=0.069 p=0.057 p=0.001 p=0.004 p=0.007

**Fig. 3:**
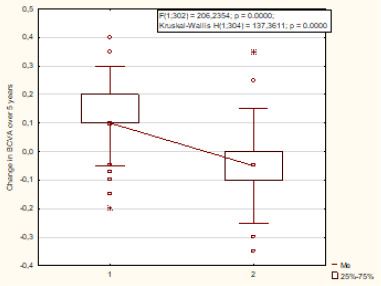
Change in BCVA (T_5_-T_0_) over 5 years of follow-up in the groups

Initial BCVA was significantly different between the groups (p=0.003). The same results were obtained when analyzing BCVA values in the case of disease progression (p=0.019). Furthermore, BCVA was significantly higher in the second group compared to the first group, with values of 0.6 (0.3-0.7) versus 0.5 (0.25-0.6), respectively. Without AMD progression, no significant difference in BCVA between the groups was observed (p=0.14). It was found that the rate of change in BCVA (T5-T0) over five years of follow-up was significantly different in both groups (p=0.000) (**Fig. 3**).

OCT-A allows imaging of the vascular network in separate fundus layers, including the choriocapillary plexus. This makes it possible to detect the progression of AMD to geographic atrophy (GA) due to loss of choriocapillary blood flow beneath atrophic areas [14]. Several OCT-A-based studies demonstrate impaired choriocapillary blood flow in patients with GA [15,16]. However, the deficiency of choriocapillary blood flow is not always synonymous with atrophy, because it can also be ischemia preceding the development of atrophy. Therefore, we used it only as an additional tool.

At five-year follow-up, we did not observe a significant difference in central retinal thickness between the two groups regardless of AMD progression (p<0.05). However, there was a considerable difference (p=0.000) in central choroidal thickness between the treatment groups.

During the follow-up period, an increase in the size and number of drusen was observed in 30.2% and 43.2% of cases in groups 1 and 2, respectively (χ^2^=5.54, p=0.186). However, the appearance of intraretinal fluid was detected in group 2 in 11.6% of eyes and in group 1 in 1.3% of eyes (χ^2^=13.4, p=0.0003). Progression to GA was observed in 6.7% of cases in group 1 and in 21.9% of patients in group 2 (χ^2^=14.22, p=0.0002). Total changes on the fundus (i.e., drusen size, appearance of confluent drusen, increase in their number, appearance of intraretinal fluid or GA) during the 5-year follow-up period showed in group 1 in 57 eyes (38.3%) and in group 2 in 119 eyes (76.8%) (χ^2^=46.24, p=0.000).

Further, clinical data of patients were analyzed to identify their association with AMD progression, such as AMD stage, BCVA before treatment and its dynamics during the follow-up period, central choroidal thickness, central retinal thickness, ophthalmoscopy data (drusen, their size, appearance of new ones, exudation, atrophic changes), as well as during nutrient administration. Spearman correlation analysis revealed a significant (p<0.05) strong correlation of AMD progression with the age of patients (r_s_=0.68), with the dynamics of fundus changes (size/number of drusen, intraretinal fluid, RPE atrophy (T_5_-T_0_) (r_s_=0.65), weak correlation with the presence of cardiovascular pathology (r_s_=0.32), and smoking (r_s_=0.24). A significant correlation of AMD progression (p<0.05) was noted with BCVA change (T_5_-T_0_) (r_s_=-0.53), with choroidal thickness (T_0_) (r_s_=-0.47), as well as central retinal thickness (T_0_) (r_s_=-0.38).

The relationship between covariates and the relative risk of AMD progression was determined using the Cox single-factor regression method (**Table 3**).

**Table 3:** Results of single-factor Cox regression analysis of the relationships of independent covariates on AMD progression

Covariate	B (SE)	HR [95% CI]	p
Gender	0.05 (0.16)	1.05 [0.77; 1.18]	0,74
Age	0.21 (0.08)	1.23 [1.06; 1.4^4^]	0.007*
Treatment group 2 (irregular intake of various nutraceuticals)	1.09 (0.18)	2.97 [2.07; 4.27]	0.000*
AMD stage (T_0_)	0.25 (0.17)	1.28 [0.9^2^; 4.7^8^]	0.143
BCVA (T_0_)	0.22 (0.31)	1.24 [0.68; 2.2^8^]	0.48
Fundus status (drusen size, RPE disorder) (T_0_)	0.20 (0.14)	1.23 [0.92; 1.6^3^]	0.16
Fundus changes (size/number of drusen, fluid, atrophy) (T_5_-T_0_)	1.42 (0.38)	4.15 [1.99; 8.6^7^]	0.000*
Change in BCVA (T_5_-T_0_)	1.06 (0.29)	2.88 [1.6^4^; 5.08]	0.000*
Central choroidal thickness (T_0_)	-0.005 (0.002)	1.00 [0.9^9^; 1.00]	0.008*
Central retinal thickness (T_0_)	-0.004 (0.002)	1.00 [0.99; 1.00]	0.03
Presence of cardiovascular pathology (T_0_)	0.40 (0.16)	1.49 [1.09; 2.03]	0.013*
Smoking (T_0_)	0.18 (0.2)	1.19 [0.80; 1.81]	0.38

Note: B = regression coefficient; SE = standard error of B coefficient; HR = hazard ratio, 95% CI = 95% confidence interval, * = significant differences (p<0.05).

Twelve covariates were analyzed in a single-factor analysis. There was no association of disease progression with gender, AMD stage, fundus status (drusen size, RPE disorder) (T_0_), baseline BCVA, central retinal thickness, or smoking.

Multivariate analysis (Cox regression)-covariates of patients with early and intermediate AMD were performed. Variables associated with AMD progression according to the results of single-factor regression were included in the multivariate Cox regression analysis in a stepwise manner. The study yielded a set of independent predictors that provided the most accurate assessment of AMD progression prognosis in our cohort (**Table 4**).

**Table 4:** Results of multivariate analysis (Cox regression) of relationships of independent covariates from factors: progression

Covariate	B (SE).	Wald criterion	p	95% confidence interval		
				Lower	HR	Upper
Age	0.20 (0.07)	0.17	**0.007**	1.01	1.22	1.41
Fundus changes (size/number of drusen, exudation, atrophy) (T_5_-T_0_)	1.02 (0.38)	7.08	**0.008**	1.31	2.78	5.92
Central choroidal thickness (T_0_)	-0.8 (0.20)	16.34	**0.000**	0.30	0.45	0.66
Cardiovascular pathology (T_0_)	0.41 (0.20)	4.29	**0.04**	1.02	1.51	2.22
Treatment group 2, with irregular intake of various nutraceuticals	-1.17 (0.20)	32.62	**0.000**	2.15	3.24	4.79
Progression of AMD	-0.81 (0.36)	4.90	**0.02**	1.10	2.24	4.57

Note: χ^2^=154.9, p=0.000. B = coefficient in Cox regression; SE = standard error for Cox regression coefficient; Wald test tests the null hypothesis that the relative risk of AMD progression associated with a given variable is equal to one; HR = hazard ratio, is the increased or decreased risk of reaching an endpoint (AMD progression) at any time during a patient's treatment with nutraceuticals, taking into account the effect of all other predictors. (HR) > 1 means increased relative risk; (HR) < 1 means decreased relative risk of reaching the endpoint during the study; 95% CI = 95% confidence interval hazard ratio, applicable to estimation of the population value of the hazard ratio.

The analysis revealed a significant association between patients’ age and disease progression, as observed in both single-factor and multivariate regression analyses (HR 1.22 [95% CI 1.01-1.41], p = 0.001), indicating a 1.22-fold increase in the relative risk of AMD progression with age.

Fundus condition (drusen size/number, RPE disorder) at the beginning of treatment (p=0.012) and the dynamics of these changes (increased drusen size/number, intraretinal fluid, GA) during the follow-up period had a significant possible risk of disease progression (HR 2.78 [95% CI 1.31-5.92]), the influence of which remained significant in multivariate Cox analysis (p=0.008).

Progression of AMD was noted in patients with thinner choroid (p=0.000). This association had a significant effect in multivariate Cox regression analysis as well (p=0.000).

There was also a distinct relative risk of AMD progression in patients with the presence of cardiovascular disease, which persisted in multivariate regression analysis (HR 1.51 [95% CI 1.02-2.2^2^], p=0.04).

In multivariate Cox regression analysis, we found that the progression of AMD over 5 years for not regularly consuming various nutraceuticals had a 3.24-fold increase in relative risk [95% CI 2.15-4.79], p=0.000) compared to the group who took the recommended nutraceutical regularly, after accounting for several clinical factors: age (p=0.007), fundus change over the follow-up period (p=0.008), central choroidal thickness (p=0.000) and the presence of cardiovascular pathology (p=0.04). The hazard ratio increased with increasing patient age (**Table 5**).

**Table 5:** Results of multivariate analysis (Cox regression): Hazard ratio of AMD progression in groups depending on their age

Age	HR [95% CI] for the 5-year follow-up period	
	Group 1	Group 2
54-59	3.46 [2.06; 5.82]	11.11 [6.41; 19.24]
60-64	4.23 [2.46; 7.26]	13.57 [7.68; 23.97]
65-69	5.16 [2.84; 9.39]	16.57 [8.88; 30.91]
70-82	6.30 [3.19; 12.47]	20.24 [10.0; 40.95]

Note: HR = hazard ratio, is the increased or decreased risk of reaching an endpoint (AMD progression) at any time during a patient’s treatment with nutraceuticals, considering the effect of all other predictors. (HR) > 1 means increased relative risk; (HR) < 1 means decreased relative risk of reaching the endpoint during the study; 95% CI = 95% confidence interval hazard ratio, applicable to estimation of the population value of the hazard ratio.

**Fig. 4:**
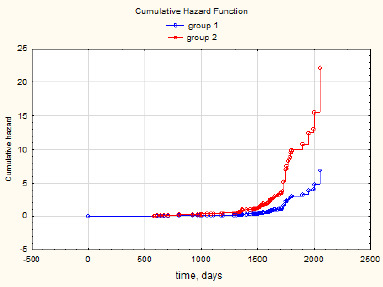
Graph showing the proportion of patients at the relative risk of AMD progression over the 5-year follow-up period, depending on the use of nutraceuticals

Furthermore, a graph illustrating the relative risk of AMD progression during the follow-up period, depending on the use of nutraceuticals, was plotted (**Fig. 4**).

## Discussion

Literature analysis of the incidence of AMD revealed an increase in this pathology. In 2050, the number of new cases of early and late AMD is projected to be 39.05 million and 6.41 million, respectively [17]. The mean unemployment rate attributed to AMD ranged from 5.50% to 77.00% [18].

According to some authors, early AMD was observed in 12.2% to 14.5% of cases, and intermediate AMD in 18.1% [19,20]. In our study, we determined the prevalence of early/intermediate AMD over five years, based on data from leading ophthalmologic centers in Ukraine. Specifically, the prevalence was 7.1% in Odesa, 6.6% in Kharkiv, and 6.3% in Moldova, which is consistent with existing literature data.

The age distribution of patients with AMD in our study was as follows: 16.1% aged 54-59, 25.3% of patients aged 60-64, 65 to 69 years - 20.1%, 70 to 82 years - 38.5%. Brandl (2018) reported that at the age of 65 to 74 years, the prevalence was 24% and up to 44% in those aged 70 to 95 years [21]. In our sample, 75.3% of patients were younger than 70 years. This is a significant social issue because active individuals are experiencing a decline in central vision, work capacity, and quality of life.

Patients with drusen <63 μm were not included in our study, because based on our experience and data by Ferris et al., these age-related changes do not have a clinically significant increased risk of developing late AMD [12]. In our sample, the initial stage of AMD was diagnosed most often, which can be explained by the fact that 68% of patients were of working age, and more often consulted an ophthalmologist when they had significant visual acuity disturbances.

In our sample, age was associated with a higher risk of AMD progression (HR 1.27 [95% CI 1.12-1.45]), a finding consistent with other authors [17,22-25]. Gender had no significant effect on disease progression, as determined by other researchers [23,24]. However, some authors have observed a sex-dependent risk of AMD progression [25,26]. In our work, we observed this relationship in the single-factor Cox regression, and further in the multivariate analysis, this relationship was not significant (p>0.05). This may be due to the uneven number of men and women in our sample (there were 1.9 times fewer men than women in the study cohort). In our sample, the initial visual acuity did not affect the progression of AMD, unlike findings from previous authors who noted this risk after two years [26]. However, in patients with disease progression, there was a negative dynamic of BCVA (p=0.000), with no significant influence of this covariate in multivariate analysis (p<0.05).

The current study showed a significant correlation between AMD progression and fundus changes (r_s_=0.65, p<0.05). The changes we observed during the follow-up period (these were drusen size/appearance of confluent drusen, increase in their number, and appearance of intraretinal fluid/GA) were, in general, significantly different in both groups (χ^2^=46.24, p=0.000) and amounted to 57 eyes (38.3%) in group 1 and 119 eyes (76.8%) in group 2. Thus, Sénéclauze and colleagues identified a 4.41-fold increased risk (95% CI, 1.98-9.81) of developing late AMD when drusen were centrally located [27]. In our sample, we did not consider the location of drusen. We thought the increase in the number of drusen and their sizes (appearance of confluent drusen), which was determined in 30.2% and 43.2% of cases in groups 1 and 2, respectively (χ^2^=5.54, p=0.186). However, the appearance of intraretinal fluid was observed in 1.3% of eyes in group 1 and in 11.6% of eyes in group 2 (χ^2^=13.4, p=0.0003), which was consistent with the results of the study without treatment (11.7%) [22]. Progression to GA in our research in the group with irregular intake of various vitamin complexes was 21.9%, which was significantly different in the group with constant use of nutraceuticals - 6.7% (χ2=14.22, p=0.0002). In another study, 11% was indicated without treatment [22]. Cicinelli and colleagues identified anatomical (thinner outer nuclear layer) and functional (worse baseline visual acuity) factors influencing the risk of foveolar lesions in GA [28]. We detected a correlation between AMD progression and central choroidal thickness (r_s_=-0.67, p<0.05) as well as central retinal thickness (r_s_=-0.43, p<0.05). Still, in further multivariate Cox analysis, we did not determine the risk of AMD progression from retinal thickness in the macula. We may not have considered the individual retinal layers, nor emphasized the macular/paramacular location of drusen. This will be promising in future studies. However, we identified a more likely progression of AMD with a thin choroid (p=0.000). This association remained significant in multivariate Cox regression analysis (p=0.000). Our data agree with the definition of the development of GA with loss of choriocapillaris and decreased blood flow in the work of Greig [29].

Some authors associate smoking with AMD development and risk of disease progression [22**-**24,27]. We observed a direct weak correlation between AMD progression and smoking (r_s_=0.24, p<0.05). However, further analysis did not determine this relationship, probably due to the small number of smokers in the sample (24%). In the study of Wang, it was determined that smoking is an independent risk factor even in advanced AMD [17]. According to this data and other studies, the risk factor was the level of cholesterol (high-density lipoprotein) [17,22,27,30], which we did not analyze in our work. However, we noted a direct, albeit insignificant, association between cardiovascular pathology and AMD progression (rs=0.32). In multivariate analysis, this association was confirmed as a risk factor for disease progression in these patients (HR 1.51 [95% CI 1.02-2.22], p=0.04).

An essential factor in the progression of AMD is the degeneration of RPE cells. A significant component of lipofuscin granules is the toxic fluorophore N-retinyl-N-retinylidene ethanolamine (A2E), which accumulates in RPE with age. Nutrients with antioxidant properties may play a potential role in both the prevention and treatment of this age-related disease. In particular, there has been increased interest in the therapeutic effects of resveratrol, which has antiproliferative, antioxidant, anti-inflammatory, and antiapoptotic effects that coincide with the pathogenetic treatment of AMD [31**-**35].

A healthy diet is recommended from the early stages of AMD, and the use of antioxidants at an intermediate stage of the disease (or even in the early stage, if the diet is not enough or is unattainable). Antioxidant-vitamin complexes have also been shown to slow progression to late AMD [36]. Thus, in our study, irregular nutraceutical intake was identified as a significant predictor in a single-factor analysis of AMD progression (HR 2.97, [95% CI 2.07; 4.27], p=0.000). After 5 years, the probable risk of AMD progression was 3.24 times lower in the group with regular intake of AREDS2 formula nutraceuticals with omega-3 PUFAs, vitamin D, and resveratrol than in the group with irregular use of various vitamin-antioxidant complexes (HR 3.24 [95% CI 2.15-4.79], p=0.000). The hazard ratio of AMD progression increased with increasing patient age. Thus, antioxidants may be effective in the treatment of AMD by delaying or reducing oxidative damage to RPE, which may reduce the progression and development of GA. The most robust clinical trial data are from the AREDS studies. Recent studies of antioxidant intake with vitamins A, C, E, zinc, omega-3 PUFAs, and resveratrol confirmed the process of scavenging free radicals and preventing or slowing the progression of AMD [37].

## Conclusion

The five-year prevalence of early and intermediate AMD was assessed using data from leading ophthalmologic centers in Ukraine (Odesa - 7.1%, Kharkiv - 6.6%) and Moldova (6.3%).

The progression of AMD in a multivariate Cox regression model over five years shows a 3.24-fold reduction in relative risk (95% CI: 2.15-4.79, p=0.000) for patients with early and intermediate AMD who regularly take the recommended nutraceutical (compared to those who irregularly take various vitamin-antioxidant complexes). This finding considers several clinical and morphological factors, including age (p=0.007), fundus changes (p=0.008), central choroidal thickness (p=0.000), and the presence of cardiovascular pathology (p=0.04).

Patients with early and intermediate AMD are advised to undergo courses of PBM every six months. Additionally, it is essential to address cardiovascular issues and consistently use the AREDS2 nutraceutical formula, which includes omega-3 PUFAs, vitamin D, and resveratrol. Following these recommendations can reduce the likelihood of disease progression by at least 3.24 times over the next five years.
